# Pharmacological Modulation of Functional Phenotypes of Microglia in Neurodegenerative Diseases

**DOI:** 10.3389/fnagi.2017.00139

**Published:** 2017-05-15

**Authors:** Gyun Jee Song, Kyoungho Suk

**Affiliations:** BK21 Plus KNU Biomedical Convergence Program, Department of Pharmacology, Brain Science & Engineering Institute, School of Medicine, Kyungpook National UniversityDaegu, South Korea

**Keywords:** pharmacological modulator, microglial polarization, neurodegenerative diseases, neuroinflammation, neuroprotective

## Abstract

Microglia are the resident innate immune cells of the central nervous system that mediate brain homeostasis maintenance. Microglia-mediated neuroinflammation is a hallmark shared by various neurodegenerative diseases, including Alzheimer’s disease, Parkinson’s disease, and multiple sclerosis. Numerous studies have shown microglial activation phenotypes to be heterogeneous; however, these microglial phenotypes can largely be categorized as being either M1 or M2 type. Although the specific classification of M1 and M2 functionally polarized microglia remains a topic for debate, the use of functional modulators of microglial phenotypes as potential therapeutic approaches for the treatment of neurodegenerative diseases has garnered considerable attention. This review discusses M1 and M2 microglial phenotypes and their relevance in neurodegenerative disease models, as described in recent literature. The modulation of microglial polarization toward the M2 phenotype may lead to development of future therapeutic and preventive strategies for neuroinflammatory and neurodegenerative diseases. Thus, we focus on recent studies of microglial polarization modulators, with a particular emphasis on the small-molecule compounds and their intracellular target proteins.

## Introduction: Diversity of Microglial Phenotypes and Disease Relevance

Microglia, the resident immune cells of the central nervous system (CNS), are highly specialized macrophages that play a fundamental role in neurodegenerative diseases such as Alzheimer’s disease (AD), Parkinson’s disease (PD), and multiple sclerosis (MS) (Mandrekar-Colucci and Landreth, 2010; Colonna and Butovsky, 2017). Microglia have been traditionally classified as either of the following: (1) resting with branched morphology and present in healthy brains or (2) activated with amoeboid morphology and present in diseased brains. Recent microglia classifications are more complex. Activated microglia are now recognized as being heterogeneous and plastic, and exist in various phenotypes in the CNS. Microglia can be divided into at least two types (neurotoxic or neuroprotective) based on their function (Kettenmann et al., 2013). Microglia can promote neurotoxicity via the release of several pro-inflammatory mediators, such as nitric oxide, interleukin (IL)-1β, and tumor necrosis factor-alpha (TNF-α) (Hanisch, 2002; Block et al., 2007). Conversely, they can be neuroprotective and neurosupportive, via several mechanisms under certain conditions. For example, neuroprotective roles of microglia include glutamate uptake (Byrnes et al., 2009), removal of dead cell debris and abnormally accumulated proteins (Diaz-Aparicio et al., 2016), and production of neurotrophic factors such as insulin-like growth factor-1 (IGF-1) (Thored et al., 2009), glial cell-derived neurotrophic factor (GDNF) (Lu et al., 2005), and brain-derived neurotrophic factor (BDNF) (Batchelor et al., 1999).

The dual nature of microglial functional polarization is consistent with the general classification of macrophages as being either the M1 (classic pro-inflammatory) or M2 (anti-inflammatory) phenotype (Michelucci et al., 2009). Specific environmental cues induce macrophages to adopt a given functionality. For example, stimulation with either lipopolysaccharide (LPS) or interferon (IFN)-γ induces activation of the classical M1 phenotype, whereas stimulation with either IL-4 or IL-13 induces the M2 activation (Cherry et al., 2014; Loane and Kumar, 2016). Microglia are critical to immune response in the CNS, and unsurprisingly, microglial functional polarization has been implicated in almost all CNS disorders, and in the progression of neurodegenerative diseases (Tang and Le, 2016). Microglia also play key functional roles in recovery from brain injury and in the maintenance of homeostasis in the brain.

### Microglia in Neurodegenerative Disease

Neurodegenerative diseases such as AD, PD, amyotrophic lateral sclerosis (ALS), and MS, are characterized by neuronal degeneration in specific regions of the CNS, sharing common pathophysiological mechanisms including inflammation and abnormal protein deposition (Heppner et al., 2015; Chen et al., 2016). AD is the most prevalent neurodegenerative disorder that is characterized by progressive focal cortical atrophy, cognitive dysfunction, β-amyloid deposition, and neuronal degeneration that initiates with neurons of the hippocampus and cortex. PD is the second-most prevalent disorder, and is characterized by a progressive loss of the dopaminergic neurons in the substantia nigra and accumulation of α-synuclein and other abnormal proteins in Lewy bodies. ALS is a chronic degenerative disease characterized by the progressive degeneration of motor neurons. The accumulation of abnormal proteins such as superoxide dismutase (SOD)1 and transactive response (TAR) DNA binding protein 43 kDa (TDP-43) in motor neurons is the major pathological feature. MS is an inflammatory and degenerative disease of the CNS characterized by autoimmunity, monocyte infiltration, microglial activation, demyelination, and neuronal death. Inflammatory mediators, such as IL-1β, IL-6 TNF-α, chemokines, matrix metalloproteinase 2 (MMP2), nitric oxide, and nuclear factor kappa-B (NF-κB), contribute to neurodegeneration and myelin damage in these neurodegenerative diseases. Activated microglia and macrophages produce these inflammatory mediators, orchestrating neuroinflammation, and neurodegeneration. Neuroinflammation is initially a protective response in the brain, however, uncontrolled inflammatory responses are detrimental as they induce the release of high levels of neurotoxic factors such as ROS and TNF-α, and block neuronal regeneration (Chen et al., 2016).

Therefore, demonstrating a microglial functional polarization would potentially uncover new prospects for understanding the way in which neurodegeneration can be promoted by local CNS inflammation. In AD, inflammation is considered to be a pathology that occurs in response to β-amyloid accumulation. Classical (M1) activation of microglia is considered the initial defense mechanism in AD, and is characterized by the release of proinflammatory cytokines. Conversely, alternative (M2) activation is associated with angiogenesis, neurogenesis, anti-inflammatory effects, and the degradation of β-amyloid deposits. Thus, modulation of microglial activation toward the M2 phenotype has been proposed as a novel therapeutic strategy for patients with AD. Indeed, Latta et al. (2015) showed that an enhanced M2 phenotype, induced either by IL-4 overexpression, or treatment with exogenous IL-4, decreased β-amyloid deposition both *in vitro* and in an animal model. Similarly, M1 polarization of microglia induced by expression of IFN-γ has been shown to increase the amyloid burden in an amyloid precursor protein/presenilin1 (APP/PS1) AD mouse model (Weekman et al., 2014).

Microglia-mediated neuroinflammation is also a hallmark of PD. In the brains of PD patients, microglia exert both neurotoxic and neuroprotective effects depending on the surrounding microenvironment. Persistent microglial activation by damaged neurons and α-synuclein deposition is generally detrimental. Reactive microglia release a range of reactive oxygen species, such as nitric oxide and superoxide-anion and pro-inflammatory cytokines, which exacerbate motor deficits in PD. Therefore, many anti-inflammatory agents have been proposed as promising PD therapeutic agents. Indeed, non-steroidal anti-inflammatory drugs and minocycline have been used in clinical studies for PD patients (Gao and Hong, 2008).

Accumulation of the misfolded ALS-linked mutant SOD1 or TDP-43 is tightly associated with the neurotoxic M1 inflammatory microglial activation (Boillee et al., 2006; Swarup et al., 2011; Huang et al., 2012). Primary microglia isolated from SOD^G93A^ transgenic mice are more neurotoxic compared to wild-type microglia, due to an increased production of superoxide and nitric oxide as well as the decreased expression of IGF-I (Xiao et al., 2007). Furthermore, IL-4-induced M2 microglia reduced LPS-induced microglia-mediated motor neuron injury (Zhao et al., 2006) and disease stage-dependent microglial switch from neuroprotective to neurotoxic phenotype has been observed in an ALS mouse model.

Microglia isolated from ALS mice at disease onset expressed higher levels of M2 markers and lower levels of the M1 marker, NADPH oxidase (NOX)2, compared with those isolated at the end-stage of ALS, indicating a diminished function of neuroprotective microglia in the late stage of the disease (Liao et al., 2012). Thus, the administration of minocycline delays the pathogenesis of SOD^G93A^ mice by selectively attenuating the induction of M1 microglia markers during the progressive phase, without affecting the transient enhancement of M2 microglia markers at the early stage (Kobayashi et al., 2013).

Microglial activation has also been studied extensively in MS patients and in the experimental autoimmune encephalomyelitis (EAE) mouse model. In this pathological condition, microglia release neurotoxic and neurotrophic molecules, pro- and anti-inflammatory cytokines, playing both beneficial and detrimental roles during the demyelination and recovery stages (Correale, 2014). Miron et al. (2013) examined whether M2 phenotypes contribute to regenerative response in the CNS. In their study, the M1 to M2 switch was observed at the initiation of remyelination, 10 days post-injection of lyso-phosphatidylcholine (lecithin). Oligodendrocyte differentiation for regeneration was enhanced by M2 microglia-conditioned medium. M2 polarization of microglia has been proposed to preserve myelin homeostasis after white matter injury in traumatic brain injury (TBI) or cuprizone-induced demyelination models (Chen et al., 2014c; Wang et al., 2015). Furthermore, the protective mechanisms exerted by alternatively activated (M2) microglia have been discussed in recent review articles (Cherry et al., 2014; Du et al., 2016; Tang and Le, 2016). Thus, enhancing the neuroprotective effects of these M2 microglia may be a promising therapeutic approach.

Prion disease is another progressive neurodegenerative disorder, and like many other neurodegenerative diseases, it is characterized by misfolded protein aggregates and neuroinflammation (Burchell and Panegyres, 2016; Stopschinski and Diamond, 2017). In prion disease, misfolded prion protein aggregates propagate by the conversion of normal cellular prion protein (PrPC) to abnormal isoforms, designated pathogenic conformers of the prion protein (PrPSc), which causes rapid neurodegeneration accompanied by spongiform change and neuronal loss in the brain. Furthermore, the crosstalk between misfolded proteins in animal models of Alzheimer’s and prion diseases has been proposed in recent studies (Morales et al., 2010; Fernandez et al., 2017), suggesting that one protein misfolding process may be an important risk factor for the development of other protein aggregation-induced diseases. Importantly, microglial proliferation, activation, and phenotype conversion have been associated with overall prion disease progression (Aguzzi et al., 2013; Grizenkova et al., 2014; Lins et al., 2016).

### Specific Markers for the M1 and M2 Phenotypes

M1-type microglia release diverse proinflammatory mediators and free radicals that inhibit brain repair and regeneration. Conversely, microglia of the M2 phenotype improve brain repair and regeneration by enhancing phagocytosis, releasing trophic factors, and reducing brain inflammation. Following stimulation with LPS or IFN-γ, M1 microglia express high levels of inducible nitric oxide synthase (iNOS) and pro-inflammatory cytokines/chemokines such as TNF-α, IL-1β, and CC chemokine ligand (CCL)2. IL-4- or IL-13-stimulated microglia express Arg1, Ym-1, CD200R, IL-10, transforming growth factor (TGF)-β and Fizzl-1, which serve as specific markers for M2 microglia. M1 and M2 microglia represent a spectrum of various activation phenotype rather than single phenotype of each status. Three subtypes of M2 microglia have been proposed, such as M2a (wound-healing and anti-inflammatory phenotype expressing CD206, Fizz-1, Arg1, Ym1), M2b (inflammation modulatory phenotype expressing IL-10, COX2) and M2c (immunosuppressive phenotype expressing CD163) (Franco and Fernandez-Suarez, 2015; Du et al., 2016). More recently, Kumar et al. (2016) proposed a mixed transitional phenotype of microglia called Mtran, co-expressing M1 makers (iNOS and IL-12) and M2 markers (TGF-β and Arg1). In their study, up to 42% of Arg1 (M2 marker)-positive cells co-express the M1 marker iNOS at 7 days post-TBI indicating a significant mixed population of microglia during recovery after injury (Kumar et al., 2016), which was confirmed in the middle cerebral artery occlusion (MCAo) model (Moretti et al., 2016). Furthermore, it is difficult to discriminate between M1 or each M2 subtype *in vitro*, and even more so *in vivo*. Recent technical advancements, including the fluorescent analysis of activation markers, cell sorting, and single-cell RNA-seq analysis, have helped to define microglia-specific genes compared to those specific to macrophages and other glia (Butovsky et al., 2014; Crotti and Ransohoff, 2016). However, M1/M2 phenotypes of microglia do not precisely match the microglial classification based on a transcriptomic analysis such as RNA-seq (Yamasaki et al., 2014). Although the binary concept of microglial M1/M2 classification has been recently debated (Ransohoff, 2016), the functional classification of microglia as being either neurotoxic (M1) or neuroprotective (M2) is a useful for illustrating the pathobiology of inflammatory and degenerative CNS disorders. Therefore, we used the M1 and M2 phenotypic classification for activated microglia in this review.

## Pharmacological Approaches for Modulating Microglial Phenotypes

Currently available medication is incapable of repairing degenerated neurons, inducing regeneration, and/or preventing further neuronal death in AD, PD, ALS, and/or MS patients. Current drugs are used to reduce the severity of disease-related symptoms by limiting the extent of neuroinflammation in patients (Glass et al., 2010). Nevertheless, although these drugs reduce symptoms, and thus increase the quality of life for these patients, they are not able to repair or regenerate damaged neurons.

Given that the balance of microglial M1/M2 phenotypes has been implicated in the pathogenesis of neurodegenerative diseases, several small-molecule compounds have recently been studied to ascertain their ability to regulate microglial functional polarization, and thereby exert neuroprotection against neurodegenerative diseases in animal models. Elucidating the underlying mechanisms of action and identifying the target proteins of these small-molecule compounds may be essential to design better chemical modulators of microglial polarization and thus effective neuroprotective drugs.

The microglial microenvironment (for example, infection, ischemic injury, β-amyloid depositions, and/or pro-inflammatory mediators such as TNF-α, IL-1β, and nitric oxide), has been shown to play a critical role in determining the microglial polarization state. Several soluble factors released from neurons and astrocytes are suggested to contribute to the determination of microglial phenotypes, as demonstrated by the M2 to M1 microglial phenotypic switch induced by exposure to cell culture medium conditioned by damaged neurons or activated astrocytes. Furthermore, many intracellular molecules are involved in controlling microglial/macrophage polarization. For example, nuclear receptors [peroxisome proliferator-activated receptor (PPAR)γ, PPARδ, retinoid X receptor (RXR)], redox signaling molecules [NOX2, hypoxia-inducible factor (HIF)-1α], NF-κB signaling molecules, and metabolic shift-mediated proteins are all known to control the phenotypic switching of microglia and macrophages (Sica and Mantovani, 2012). Thus, potential exploitation of these molecules may be a promising method for developing novel therapeutic drugs for neurodegenerative diseases.

Gene therapies, including BDNF- or IL4-overexpressing viruses, recombinant proteins such as IL-4, IL-10, IL-13, and/or TGF-β, Etanercept (a TNF-α-antagonist fusion protein), and cell therapies (M2 microglia and macrophages) have been applied as therapeutic tools. However, due to the blood–brain barrier, these therapeutic strategies have limited efficacy, leading to the screening of small synthetic chemical and/or natural compounds as treatments for various brain diseases. In this review, we focus on the effect and the target proteins of these small-molecule compounds that modulate M1/M2 microglial polarization (**Table 1**).

**Table 1:** Pharmacological tools for modulating microglial polarization.

Targets	Small-molecules	Models	Effects	Reference
**Nuclear receptors**				
PPARγ (agonist)	Pioglitazone	AD mice	M1 to M2 switch, reverse cognitive deficits	Toba et al., 2016
	DSP-8658	AD mice	Enhanced microglial phagocytosis, improved memory	Yamanaka et al., 2012
	MDG548	PD mice *in vitro*	Neuroprotection, reduction of NF-κB activation	Lecca et al., 2015
	SNU-BP	LPS model	Increased M2 and decreased M1	Song et al., 2016
RXR (agonist)	Bexarotene	AD mice and *in vitro*	Clearance of β-amyloid, reverse cognitive deficits	Cramer et al., 2012

**Metabolism-associated molecules**				

AMPK (activator)	Metformin	MCAo PD mice	M2 polarization, angiogenesis, neurogenesis, improved locomotor activity	Jin et al., 2014; Patil et al., 2014
PDK (inhibitor)	DCA	CFA inflammation model, *in vitro*	M1/M2 switch, reduced pain and inflammation	Jha et al., 2015
Aldose reductase (inhibitor)	Fidarestat (SNK-860)	SCI model *In vitro*	M2 polarization Reduced LPS-induced inflammation	Zhang et al., 2016; Reddy et al., 2010

**cAMP-dependent pathway for M2 polarization**		
PKA (activator)	Db-cAMP	SCI model	Induce M2 phenotype	Ghosh et al., 2016
PDE4 (inhibitor)	Rolipram PDE4D-NAM	Aged mice and primates	Neuroprotective, anti-inflammatory Memory consolidation	Barad et al., 1998; Burgin et al., 2010
PDE5 (inhibitor)	Sildenafil	MCAo AD mice EAE model	M1/M2 modulation Clearance of β-amyloid prevent axonal loss promote re-myelination	Puzzo et al., 2009; Pifarre et al., 2011; Moretti et al., 2016

**Redox signaling molecules for microglial M1/M2 balance**		

NOX (inhibitor)	Apocynin	*In vitro* PD mice	Induce M2 polarization protect from dopaminergic neuron degeneration	Choi et al., 2012; Hernandes et al., 2013a
	GKT137831	Diabetic retinopathy, *in vitro*	Hypoxia-induced ROS production and the expression of inflammatory cytokines in retinal microglia	Deliyanti and Wilkinson-Berka, 2015

**M1 polarization for inhibiting glioma cell growth**		

mTOR (inhibitor)	Rad	Glioma	M1 polarization, prevented glioma growth	Lisi et al., 2014
–	Chlorogenic acid	Glioma	Increased M1 and decreased M2 reduction of tumor size	Xue et al., 2017

**Other signaling molecules**		

HDAC (inhibitor)	Scriptaid	TBI *In vitro* model	M2 polarization, protect white brain matter, suppress LPS-induced cytokine expression	Wang et al., 2015; Kannan et al., 2013
ROCK (inhibitor)	FSD-C10 Y-39983	EAE model EAE model	Inhibit neuroinflammation Attenuation of demyelination	Li et al., 2014; Gao et al., 2013
	Fasudil	*In vitro*	M2 polarization	Chen et al., 2014a

*AD, Alzheimer’s disease; PD, Parkinson’s disease; MS, multiple sclerosis; MCAo, middle cerebral artery occlusion; SCI, spinal cord injury; TBI, traumatic brain injury; EAE, experimental allergic encephalomyelitis; CFA, complete Freund’s adjuvant; LPS, lipopolysaccharide; PDE4D-NAM, PDE4D negative allosteric modulator.*

### Nuclear Receptors

The nuclear hormone receptor PPAR is a key regulator of the M2 phenotype in macrophages and microglia (Bouhlel et al., 2007; Chawla, 2010). PPAR activation increases phagocytic uptake of amyloid-β plaques, and is neuroprotective in an AD mouse model (Mandrekar-Colucci et al., 2012; Yamanaka et al., 2012). Accordingly, treatment with the PPARγ agonist, pioglitazone, results in the phenotypic switch of proinflammatory M1 to anti-inflammatory M2 microglia. Furthermore, pioglitazone treatment dramatically reduces the levels of soluble and insoluble β-amyloid, and reverses the cognitive deficits in 12-month-old APP/PS1 mice (Toba et al., 2016). A novel selective-PPAR modulator, DSP-8658, was shown to enhance the microglial phagocytosis and improve the spatial memory performance in an AD mouse model (Yamanaka et al., 2012). Similarly, a PPARγ agonist called rosiglitazone has also been shown to induce IL-4 expression in the rat brain as well as age-dependent M1 microglial activation in an IL-4 dependent manner (Loane et al., 2009). A novel compound, MDG548, was found to induce neuroprotection in MPTP-treated mice and decrease LPS-induced NF-κB activation in a dose-dependent manner (Lecca et al., 2015). Finally, an *N*-carbamolylated urethane compound (SNU-BP) has been identified as a novel PPARγ agonist, and inhibits LPS-induced pro-inflammatory cytokine and nitric oxide production as well as potentiates IL-4-induced M2 marker expression in microglia and astrocytes. SNU-BP was also shown to exhibit an anti-neuroinflammatory effect in an LPS-injected mouse model, probably via the M1/M2 switch (Song et al., 2016).

Alzheimer’s disease (AD) is strongly associated with the impaired clearance of β-amyloid from the brain. The RXR agonist bexarotene (Targretin) has been shown to enhance β-amyloid clearance by activating PPARγ/RXR and liver X receptors (LXRs)/RXR, inducing apoE expression, and promoting microglial phagocytosis (Cramer et al., 2012). It is a highly selective, synthetic retinoid analog with specific affinity for the RXR, which is able to cross the blood–brain barrier and has a favorable FDA-approved safety profile (Skalak et al., 1987; Cramer et al., 2012).

Malibatol A (MA) is a natural resveratrol oligomer extracted from the leaves of the Chinese plant *Hopea hainanensis*. MA has an anti-inflammatory effect on MCAo mice and LPS-stimulated microglia. MA treatment was found to decrease the expression of M1 (CD16, CD32, and CD86), while increasing that of M2 markers (CD206 and YM-1), in a PPARγ-dependent manner (Pan et al., 2015).

### Metabolism-Associated Proteins

Evidence suggests a role for metabolic reprogramming in the modulation of M1/M2 microglial phenotypes (Orihuela et al., 2016). Various studies support the belief that mitochondrial metabolic shifts are associated with microglial polarization. Under normoxic conditions, ATP (energy) production is achieved via oxidative phosphorylation; in contrast, anaerobic glycolysis converts pyruvate into lactate under hypoxic conditions. In the context of the peripheral immune cells, a shift in the cellular metabolism from oxidative phosphorylation to aerobic glycolysis favors the polarization of microglia toward the M1 phenotype. This metabolic switch is promoted by PI3K/AKT and inhibited by AMP-activated protein kinase (AMPK) and IL-10.

AMP-activated protein kinase activation is associated with neuroprotection after stroke (Jin et al., 2014; Venna et al., 2014) via enhanced neurogenesis and lowered blood glucose levels. The AMPK activator metformin promotes both functional recovery and tissue repair following stroke. Mice that administered metformin daily after MCAo exhibited more M2-polarized microglia and macrophages, as well as increased angiogenesis and neurogenesis (Jin et al., 2014). The same study also demonstrated metformin-induced M2 polarization of BV-2 microglial cells dependent upon AMPK activation. Patil et al. (2014) similarly demonstrated the neuroprotective effect of metformin in a PD mouse model, such that impaired locomotor activities and the loss of TH-positive cells were both significantly improved in metformin (500 mg/kg for 21 days)-treated PD mice, compared to controls.

Pyruvate dehydrogenase kinases (PDKs) are mitochondrial metabolic regulators that modulate pyruvate dehydrogenase (PDH) activity to convert pyruvate either aerobically to acetyl-CoA, or anaerobically to lactate (Jha et al., 2015). *In vitro* studies support the role of PDK2/4 as promoters of the classical proinflammatory (M1) activation of macrophages. Moreover, the pharmacological PDK inhibitor dichloroacetate (DCA) diminishes complete Freund’s adjuvant (CFA)-induced inflammation and pain via an induced M1-M2 switch (Jha et al., 2015). This suggests that a pathological metabolic shift, and subsequent lactic acid production, each contribute to neuroinflammatory disease progression.

Fidarestat (SNK-860) is an aldose reductase (AR) inhibitor used for the treatment of diabetic neuropathy (Kuzumoto et al., 2006). A recent study showed the incubation of macrophages with LPS to cause a significant increase (6-fold) in the uptake of glucose in a time dependent manner. In contrast, the inhibition of AR via fidarestat treatment decreased LPS-induced glucose uptake by 30% (Reddy et al., 2010). AR expression is enhanced in microglia/macrophages after spinal cord injury (SCI). The study further demonstrated that AR inhibition and/or deficiency in microglia/macrophages favors a phenotype switch toward the M2, rather than the M1 phenotype, and that AR inhibition induces the phosphorylation of cAMP response element-binding protein (CREB) leading to the enhanced expression of the M2 marker, Arg-1 (Zhang et al., 2016).

### cAMP-Dependent Pathways and their Regulators

Cyclic adenosine monophosphate (cAMP) is a well-known regulator of microglial function and activation (Ghosh et al., 2012, 2015, 2016). The elevation of cAMP in microglia or macrophages via the application of an adenylyl cyclase activator, synthetic cyclic AMP analoges or phosphodiesterase (PDE) inhibitors, inhibits the production of pro-inflammatory molecules (Gerlo et al., 2011). Interestingly, proinflammatory cytokines such as TNF-α and IL-1β both rapidly reduced cAMP, and increased PDE4 expression in microglia (Ghosh et al., 2012). The protective effect of cAMP on neuronal regeneration has been reported in rat SCI (Neumann et al., 2002) and cerebral ischemia-reperfusion injury models (Niu et al., 2010). Dibutyryl (db)-cAMP is a membrane-permeable derivative of cAMP that exhibits a prolonged response time. Ghosh et al. (2016) investigated the effect of cAMP on the modulation of microglial phenotypes and found that co-treatment of microglial cells with cAMP and IL-4 induced an M2 phenotype (Arg-1+/iNOS-) with concomitant expression of various M2-specific markers including TG2 and FIZZ1. Administration of db-cAMP and IL-4 also promoted M2 phenotypes in the lesions of SCI model mice (Ghosh et al., 2016). Together, these data strongly support that cAMP is a critical determinant of M1-M2 polarization.

PDE4 is an enzyme that negatively regulates cAMP signaling by hydrolyzing cAMP in immune and brain cells. PDE4 inhibitors (including rolipram) are reported to have precognitive, neuroprotective, and anti-inflammatory effects (Barad et al., 1998; Dinter, 2000; Block et al., 2001). PDE4-negative allosteric modulators (NAMs) have been recently developed for specific inhibition of each PDE4 subtype (i.e., PDE4A, B, C, and D). PDE4D-NAM has been shown to improve cognitive performance in healthy rodents (Burgin et al., 2010) and primates (Sutcliffe et al., 2014). PDE4D-NAMs (including D158681, D159153, D159404, and D159687) have also been demonstrated to produce potent cognitive benefits by augmenting signaling via the cAMP/protein kinase A/CREB pathway for memory consolidation (Burgin et al., 2010; Gurney et al., 2015).

PDE4 subtypes are known to modulate the inflammatory response in the brain, such that TNF-α increases PDE4B expression and nuclear translocation in microglia (Ghosh et al., 2012). Of the PDE4 subtypes, PDE4B in particular is highly expressed in activated microglia after TBI and SCI. Importantly, this observation promotes the potential application of therapeutic modulators for the treatment of neuroinflammatory diseases including AD.

PDE5 inhibitor sildenafil has been recently developed as a first-line drug for diabetic patients with erectile dysfunction. Recently PDE5 inhibitors have been proposed as potential therapeutic agents for neuroinflammatory, degenerative, and memory-loss diseases including AD, PD, and MS (Puzzo et al., 2009; Pifarre et al., 2011; Fiorito et al., 2013; Peixoto et al., 2015). Possible underlying mechanisms for the beneficial effects of PDE5 inhibitors include a neuroprotective effect exerted via the cGMP and/or cAMP signaling pathways and an anti-inflammatory-related effect. In an *in vitro* study of microglia, PDE5 inhibitors were shown to inhibit LPS-induced M1 polarization by decreasing the production of nitric oxide, TNF-α, and IL-1β (Zhao et al., 2016). M2 polarization induced by sildenafil has been shown to provide protection against lesion extension in the late phase of MCAo in neonatal mice (Moretti et al., 2016). Furthermore, the chronic inhibition of PDE-5 has been shown to facilitate the shift from classic (M1) to alternative (M2) macrophage polarization in streptozotocin-induced diabetic mice (Venneri et al., 2015).

### Redox Signaling Molecules

Activated microglia-derived oxidative stress is implicated in numerous CNS diseases. The redox status modulates the acquisition of classical microglia activation phenotypes by various mechanisms, including the NOX and NOS-dependent pathways (Bermudez et al., 2016; Haslund-Vinding et al., 2016; Seredenina et al., 2016; Vilhardt et al., 2016). NOX induces M1 polarization of microglia, and thus unsurprisingly, either a NOX inhibitor (apocynin) or genetic depletion has been reported to induce M2 polarization. Choi et al. (2012) showed that pharmacological inhibition of NOX changed microglia from an M1 to an M2 phenotype. Moreover, NOX2 gene-deficient mice (gp91phox^-/-^) have been found to be completely protected against glial M1 over-activation and dopaminergic neuron degeneration in the 6-hydroxydopamine (6-OHDA)-induced PD mouse model (Hernandes et al., 2013a,b). GKT137831 (NOX1 and NOX4 inhibitor), originally developed for diabetic nephropathy, has been shown to significantly reduce both hypoxia-induced ROS production, and the expression of inflammatory cytokines in retinal microglia (Deliyanti and Wilkinson-Berka, 2015), suggesting that NOX inhibitors may be promising therapeutic agents for diabetic retinopathy and/or neuropathy.

Resveratrol (3,5,4′-trihydroxy-*trans-*stilbene) is a natural phytoalexin found in grape-skin that exerts anti-inflammatory and antioxidant, as well as various other biological effects. Recently, resveratrol has been suggested to have neuroprotective effects against many neurological diseases (Porro et al., 2015; Abdel-Aleem et al., 2016; Ghaiad et al., 2016; Jeong et al., 2016; Shi et al., 2016; Xu et al., 2017), and several underlying mechanisms for this effect have been suggested. For example, resveratrol attenuates hypoxia-induced neurotoxicity by inhibiting microglial activation (Zhang et al., 2015), and similarly constrains amyloid-β-induced microglial activation via NOX (Yao et al., 2015).

### mTOR Inhibitors and Chlorogenic Acid: M1 Polarization for Inhibiting Glioma Cell Growth

The mammalian target of rapamycin (mTOR) is a serine/threonine protein kinase involved in many cellular processes such as transcription and translation, ribosomal biogenesis, energy metabolism, and autophagy (Laplante and Sabatini, 2012). The inhibition of mTOR has been recently suggested to prevent glioblastoma, the most common and aggressive primary CNS tumor-type, originating from glial cells (Rolle et al., 2012). Rad, an inhibitor of mTOR, was recently found to significantly increase iNOS expression, and to reduce IL-10 expression, suggesting that Rad prevents the acquisition of an M2 phenotype in response to glioma factors promoting classic M1 activation. Thus, mTOR inhibition prevented glioma growth by inhibiting M2 polarization of microglial cells, thereby increasing their anti-tumor cytotoxic potential (Lisi et al., 2014).

Chlorogenic acid, a phenolic compound extracted from natural products, has been shown to exert anti-tumor effects on multiple malignant tumors (Bandyopadhyay et al., 2004; Belkaid et al., 2006). Previous studies on the anti-tumor effects of chlorogenic acid focused on its direct cytotoxic or antioxidant effect (Lewandowska et al., 2016); however, a recent report suggested a novel anti-tumorigenic mechanism for the chlorogenic acid-induced effects, in which it promotes M1 polarization to inhibit tumor growth (Xue et al., 2017). In glioma xenograft mice, chlorogenic acid was shown to increase CD11-positive M1 and decrease CD206-positive M2 cells in tumor tissues, leading to a reduction of the overall tumor size.

### Epigenetic Regulators and Other Signaling Molecules

Previous studies have shown that histone deacetylase (HDACs) inhibitors preferentially promote the transcription of neuroprotective genes, and protect against TBI-induced neuronal damage (Lazo-Gomez et al., 2013; Wang et al., 2013) and strongly suppress LPS-induced cytokine expression and release by microglia (Kannan et al., 2013). Recently, Wang et al. (2015) showed that a novel HDAC inhibitor, scriptaid, protected white brain matter from damage by TBI via polarization of microglia/macrophages toward the beneficial M2 phenotype. Scriptaid induced the expression of microglial glycogen synthase kinase 3 beta (GSK3β) and the microglial phenotypic switch from M1 to M2. Media conditioned by Scriptaid treated-microglia conferred a greater protective effect against oxygen and glucose deprivation-induced cell death in oligodendrocytes, as compared to media conditioned by vehicle-treated microglia (Wang et al., 2015).

Rho-associated kinase (ROCK) has been proposed as another potential regulator of the microglial phenotype. ROCK is a serine/threonine kinase and a key regulator in controlling formation of the actin cytoskeleton, as well as cell motility, and cell adhesion. Selective ROCK inhibitors, such as FSD-C10 or Y-39983, have been shown to exhibit therapeutic potential in an EAE model via attenuation of demyelination and neuroinflammation (Gao et al., 2013; Li et al., 2014). More recently, microglial treatment with a ROCK inhibitor Fasudil induced alteration in microglial phenotype polarization and functional plasticity, shifting the M1 to a M2 phenotype (Chen et al., 2014a).

## Conclusion

Here, we summarized the current research on pharmacological modulators of microglial phenotypes and their cellular targets. We focused on the effects of these modulators both *in vivo*, and in *in vitro* models of neurodegenerative diseases. We divided the cellular targets into groups according to their original functions, these being nuclear receptors, metabolism-associated proteins, proteins regulating the cAMP pathway, redox signaling molecules, and others. These target proteins are potential therapeutic candidates for the effective treatment of neurodegenerative diseases. Furthermore, an improved characterization of target protein functions will enable the design of novel pharmacological compounds to modulate cytotoxic and/or neurotrophic microglial phenotypes at specific stages of neurodegenerative disease. This is owing to the time-dependent microglial activation in the switch from neuroprotective to neurotoxic profiles in chronic diseases. Thus, further studies are essential in cultured cells and animal models, as well as in human patients, to enable the translation of these preliminary findings into clinically applicable interventions for AD, PD, MS, and other neurodegenerative diseases.

## Author Contributions

Both authors have made a substantial intellectual contribution to this work, and approved submission of the manuscript. GS and KS formulated the focus of this review focus. GS conducted the literature review and summarized the discussed studies. KS evaluated the manuscript and contributed to the final version.

## Conflict of Interest Statement

The authors declare that the research was conducted in the absence of any commercial or financial relationships that could be construed as a potential conflict of interest.
